# Epigenome-wide association study of peripheral immune cell populations in Parkinson’s disease

**DOI:** 10.1038/s41531-023-00594-x

**Published:** 2023-10-31

**Authors:** Maren Stolp Andersen, Ingvild Sørum Leikfoss, Ina Skaara Brorson, Chiara Cappelletti, Conceicao Bettencourt, Mathias Toft, Lasse Pihlstrøm

**Affiliations:** 1 Department of Neurology, Oslo University Hospital, Oslo, Norway; 2 Institute of Clinical Medicine, University of Oslo, Oslo, Norway; 3 Department of Neurodegenerative Disease and Queen Square Brain Bank for Neurological Disorders, Queen Square Institute of Neurology, University College London, London, UK

**Keywords:** Parkinson's disease, Epigenetics, Neuroimmunology

## Abstract

Understanding the contribution of immune mechanisms to Parkinson’s disease pathogenesis is an important challenge, potentially of major therapeutic implications. To further elucidate the involvement of peripheral immune cells, we studied epigenome-wide DNA methylation in isolated populations of CD14^+^ monocytes, CD19^+^ B cells, CD4^+^ T cells, and CD8^+^ T cells from Parkinson’s disease patients and healthy control participants. We included 25 patients with a maximum five years of disease duration and 25 controls, and isolated four immune cell populations from each fresh blood sample. Epigenome-wide DNA methylation profiles were generated from 186 samples using the Illumina MethylationEpic array and association with disease status was tested using linear regression models. We identified six differentially methylated CpGs in CD14^+^ monocytes and one in CD8 + T cells. Four differentially methylated regions were identified in monocytes, including a region upstream of *RAB32*, a gene that has been linked to *LRRK2*. Methylation upstream of *RAB32* correlated negatively with mRNA expression, and *RAB32* expression was upregulated in Parkinson’s disease both in our samples and in summary statistics from a previous study. Our epigenome-wide association study of early Parkinson’s disease provides evidence for methylation changes across different peripheral immune cell types, highlighting monocytes and the *RAB32* locus. The findings were predominantly cell-type-specific, demonstrating the value of isolating purified cell populations for genomic studies.

## Introduction

Parkinson’s disease (PD) is a progressive neurodegenerative disorder, for which there is currently no effective disease-modifying therapy. Over the last decades, a growing body of evidence has demonstrated the important role of the immune system in PD pathogenesis, yet the specific mechanisms involved and the interrelations between microglial and peripheral immune processes are incompletely understood^1,2^. The prospect of immunomodulation is currently one of the most promising strategies for disease modification in PD^3^, and an improved understanding of the contribution of different classes of leukocytes may be pivotal for further progress.

Highlighting the relevance of the adaptive immune system, T cells have been shown to infiltrate the substantia nigra^4,5^ and recognize peptides of the neuropathological hallmark protein α-synuclein presented by human leukocyte antigen (HLA) class II proteins^6^. Levels of α-synuclein autoantibodies have also been studied with mixed results^7^, and genome-wide association studies (GWAS) have consistently reported a significant PD risk signal in the HLA region on chromosome 6^8,9^. However, studies integrating genetic association statistics with cell-specific data have primarily linked genetic PD risk to innate immunity and the myeloid lineage^10–13^, a finding that is also mirrored in Alzheimer’s disease (AD)^14^. In line with these observations, a recent transcriptomic study of CD14^+^ monocytes reported widespread alterations in PD^15^.

Epigenetics influence complex disease by mechanisms of gene regulation that are partially dynamic over time, cell-type-specific, and shaped by both genetic and environmental factors^16^. A growing body of evidence implicates epigenetic mechanisms in neurodegeneration and PD pathogenesis^17–19^. DNA methylation at CpG dinucleotides is the most studied epigenetic modification in the context of complex disease. Hypothesis-free epigenome-wide association studies (EWAS) have been performed in a small series of post-mortem human brain tissue comparing PD to control brains^20–22^ and a larger study of bulk cortical tissue from our group investigated Lewy body pathology as outcome, including donors with both PD and dementia with Lewy bodies^23^. Two PD EWAS have studied isolated cortical neurons using either flow cytometry^24^ or magnetic-activated cell separation^25^. A number of PD studies have investigated differential methylation in whole blood^26–30^. Sample sizes of whole blood PD EWAS have been gradually increasing, and the largest study to date included more than 2000 participants and reported a hypermethylated CpG site in PD near *SLC7A11*, a gene involved in glutamate signaling^30^. Methylation analyses in blood may be particularly interesting as a tool to characterize immune mechanisms in PD, and a recent EWAS study highlighted immune dysregulation both in PD generally and specifically in patients with depression^29^. These are promising findings, yet the use of whole blood for differential methylation analyses entails major caveats. Whole-blood EWAS typically applies an algorithm to adjust for estimated cell-type composition, but essentially measures the noisy, joint effect of methylation across all cell types in the sample, limiting both the power to detect cell-type-specific signals and the interpretation of positive findings. Furthermore, most studies have included patients many years from PD diagnosis, when downstream effects of the disease and its treatment may have a major impact on methylation.

To further elucidate the contribution of different classes of immune cells and identify cell-type-specific alterations in gene regulation, we performed epigenome-wide DNA methylation profiling of purified cell populations representing major peripheral cell types of the innate and adaptive immune system, namely CD14^+^ monocytes, CD19^+^ B cells, CD4^+^ T helper cells and CD8^+^ cytotoxic T cells in 25 patients with early PD and 25 healthy controls. We nominate differentially methylated CpGs and regions, with monocytes showing the highest number of significant findings.

## Results

Patients were diagnosed based on Movement Disorders Society criteria^31^ and had a maximum disease duration of five years from motor onset at the time of blood draw. Eight out of 25 PD patients were included at the time of diagnosis and had not used any medication for PD. Mean disease duration from the time of PD diagnosis was 1.2 years and mean age at diagnosis was 55. Controls had no immunological nor neurological disease and no parkinsonism in first-degree family members. Demographics are presented in Table 1.

**Table 1:** Sample demographics.

	PD patients, *N* = 25	Healthy controls, *N* = 25	*P* value
Sex Female:male	12:13	15:10	0.57
Age Mean (SD)	57 (10)	58 (12)	1
Body mass index Mean (SD)	25.2 (3.5)	24.3 (2.4)	0.30
Regular smoker Never:ever	24:1	23:2	1
Age at diagnosis Mean (SD)	55 (11)	–	–
PD duration from diagnosis Mean (SD)	1.2 (1.1)	–	–
MDS-UPDRS III Median (interquartile range)	11 (9–16)	–	–
Hoehn & Yahr stage Median (interquartile range)	2 (1–2)	–	–
Levodopa-equivalent daily dose Median (Interquartile range)	300 (0–500)	–	–

*PD* Parkinson’s disease, *SD* standard deviation, *MDS-UPDRS* Movement Disorders Society Unified Parkinson’s Disease Rating Scale III.

The table summarizes the demographics of the study participants. *P* values for significant differences between the PD and control group were obtained by chi-square test for sex and smoking and *T* test for age and body mass index.

### Visualizing overall methylation patterns across cell types

**Fig. 1:**
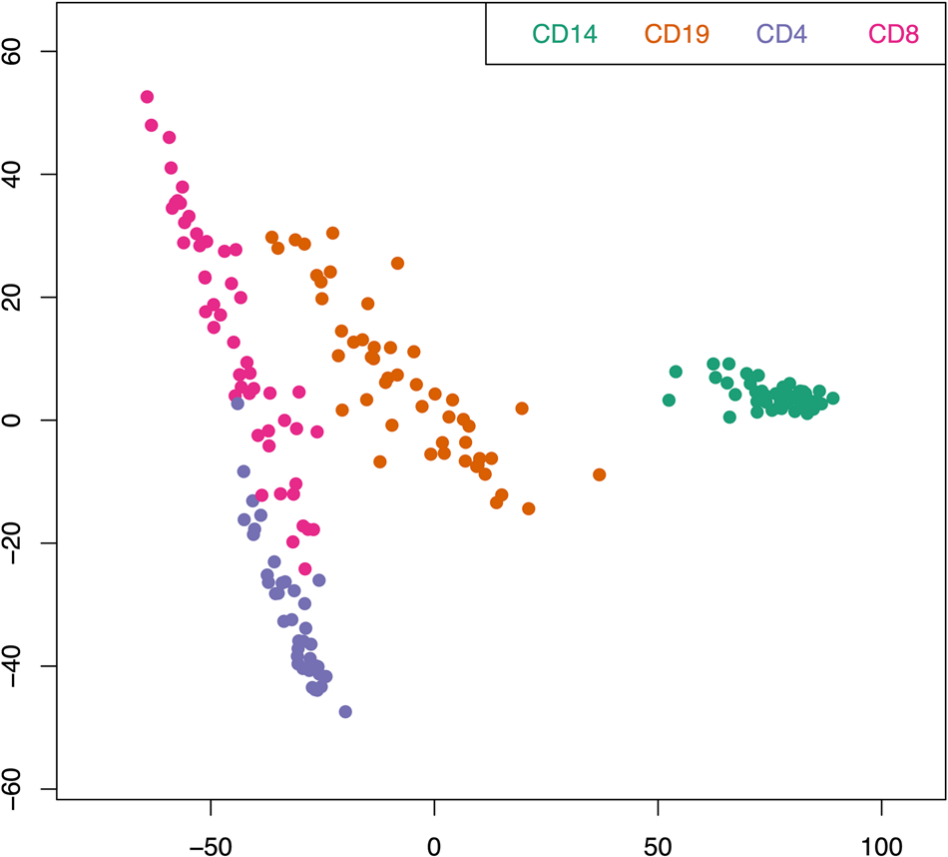
Multidimensional scaling plot. The plot visualizes the overall similarities in methylation profiles across CD14^+^ monocytes, CD19^+^ B cells, CD4^+^ T cells and CD8^+^ T cells. It was generated based on the 100,000 most variable probes using the plotting function implemented in the minfi package.

After quality control and filtering (see “Methods” and Supplementary Fig. 1), the final normalized methylation dataset included 482,470 probes and 186 samples. As expected, a multidimensional scaling plot demonstrates that the overall methylation pattern separates the CD14^+^ monocytes from the three classes of lymphocytes (Fig. 1). Within the lymphocyte group, CD4^+^, and CD8^+^ T cells cluster closer to each other than to CD19^+^ B cells.

### Power analyses and distribution of *P* values

Power analyses using pwrEWAS^32^ indicated that for a study with 25 patients and 25 controls, the difference in methylation level would need to be 0.1 SD in order for statistical power to be 80% at a false discovery rate 0.05. This corresponds to a larger effect size than many differential methylation signals reported in complex disease EWAS. We therefore expected statistical power to be marginal, although the use of purified cell populations could limit the variance and improve power substantially compared to whole-blood EWAS of similar sample size.

Following association testing in each cell type by linear regression, including sex, age, and the first surrogate variable as covariates (see “Methods”), we assessed the distribution of *P* values by quantile–quantile plots and *P* value histograms^33^ (Supplementary Figs. 2 and 3). Assuming an adequate model and true association with disease for a subset of probes, we would expect a test-statistic inflation parameter *λ* close to 1 and a uniform distribution of *P* values with an enrichment in the lower end representing the true signals. This pattern was seen most clearly in CD14+ monocytes (*λ* = 1.03), with a slight overabundance of low *P* values also in CD8 + T cells.

In particular CD4 + T cells showed skewed *P* value distributions which could indicate poor modeling of data^33^. Previous EWAS studies in both PD^25^ and Alzheimer’s disease^34^ have reported striking sex differences, arguing for sex-stratified analysis of methylation data rather than merely inclusion of sex as a covariate in linear regression. We considered our sample size too small to attempt the identification of specific differentially methylated CpGs in sex-stratified analyses, yet we performed linear regression split by sex to evaluate *P* value distributions. Interestingly, sex-stratified regression improved the pattern of *P* value distributions for both CD8 + T cells and CD19 + B cells, but not for CD14+ monocytes or CD4 + T cells. This may indicate that sex-stratified analysis is warranted for specific cell types and should be explored in future larger studies.

### Differentially methylated CpGs

**Fig. 2:**
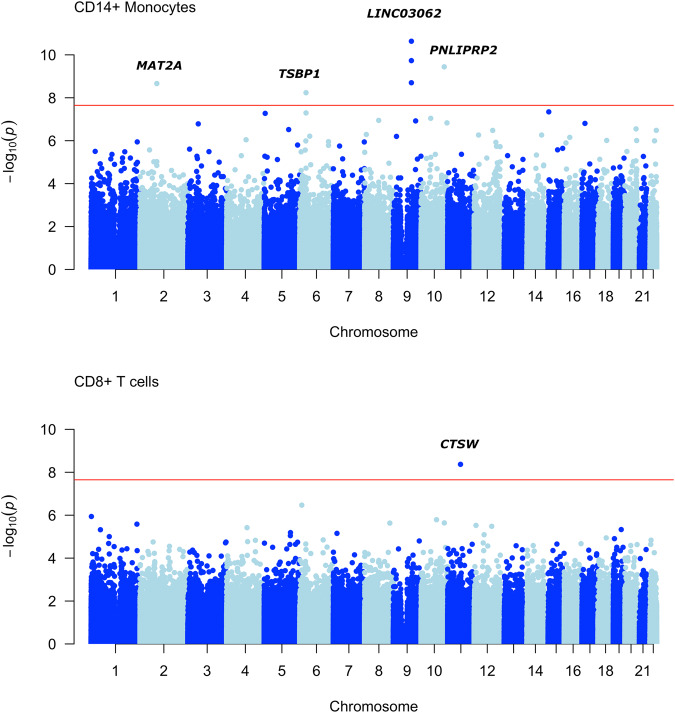
Manhattan plots. The figure shows Manhattan plots from linear regression for CD14^+^ monocytes and CD8^+^ T cells. Loci with one or more significant probes are annotated with the name of the gene closest to the top associated probe.

Association testing in linear regression models including sex, age, and the first surrogate variable as covariates (see “Methods”) identified a total of seven signals passing a Bonferroni-corrected significance threshold of *P* < 2.25 × 10^-8^ (Table 2), including six signals from four different genomic loci in CD14^+^ monocytes and one signal in CD8^+^ T cells. No significant signals were detected in CD4^+^ T cells or CD19^+^ B cells. Manhattan plots for the cell types with significant findings are shown in Fig. 2.

**Table 2:** Differentially methylated probes.

Cell type	Probe	Chr	Position	Annotated or closest* gene	Context	Coefficient (SE)	*P* value
CD14	cg13640690	chr9	92277007	*LINC03062*	Intronic	−0.68 (0.10)	2.33E-11
CD14	cg12134806	chr9	92276903	*LINC03062*	Intronic	−0.68 (0.10)	1.86E-10
CD14	cg11473614	chr10	118381374	*PNLIPRP2*	Intronic	0.44 (0.07)	3.61E-10
CD14	cg07047360	chr9	92277266	*LINC03062*	Intronic	−1.03 (0.16)	2.00E-09
CD14	cg18523915	chr2	85728591	MAT2A*	Enhancer 37.5 kb upstream	−1.98 (0.32)	2.19E-09
CD14	cg14704780	chr6	32305106	*TSBP1*	Intronic	0.72 (0.12)	5.83E-09
CD8	cg03887787	chr11	65647532	*CTSW*	Intronic	−1.03 (0.17)	4.95E-09

*Chr* chromosome, *SE* standard error.

The table shows probes passing a Bonferroni-corrected significance threshold in linear regression models contrasting Parkinson’s disease patients and controls in each cell type separately. RefSeq gene names are used, alternative names include *UNQ6494* for *LINC03062* and *C6orf10* for *TSBP1*.

*The probe cg18523915 is not annotated to any gene, the closest gene being *MAT2A*.

**Fig. 3:**
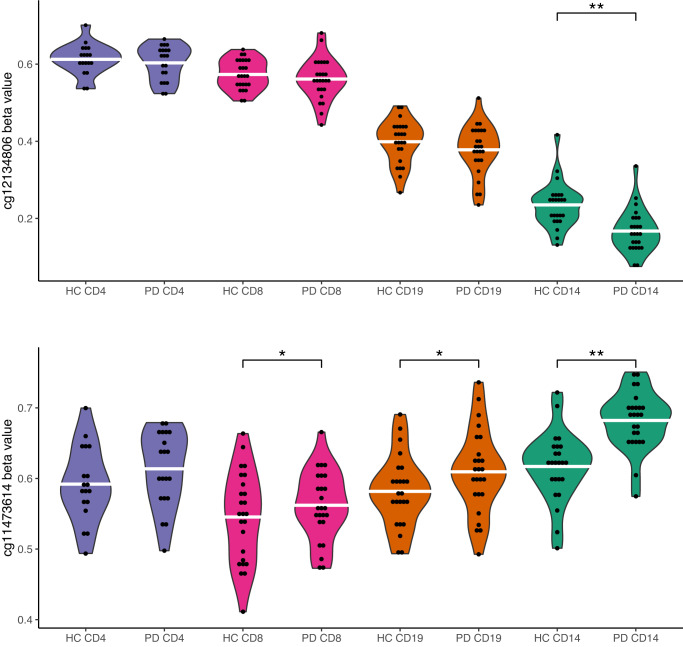
Violin plots of cg12134806 and cg11473614 methylation. The figure shows unadjusted methylation beta values across groups for probes cg12134806 and cg11473614, illustrating the contrast between a cell-type-specific and shared pattern of differential methylation. Cg12134806 shows minimal variation for all other cell types except CD14^+^ monocytes. Cg11473614 reached genome-wide significance in CD14^+^ monocytes, yet with similar directions of effect for CD19^+^ B cells (*P* = 0.0021) and CD18^+^ T cells (*P* = 0.020). White bars represent group means. Single asterisk (*) denotes *P* value < 0.05 and double asterisks (**) denotes *P* value < 2.25 × 10^–8^ in linear regression analysis, including sex, age, and one surrogate variable as covariates.

To further assess whether these association signals were cell-type-specific or shared, we extracted summary statistics from the seven significant probes for all cell types (Supplementary Table 1). A nominal association (*P* < 0.05) was seen in at least one other cell type for four out of the seven probes, but no probe showed association at *P* < 0.05 across all cell types. Associations in additional cell types were generally much weaker than the top cell type, and only one passed a Bonferroni-corrected threshold for a total of 21 candidate tests (cg11473614, genome-wide significant in monocytes, *P* = 0.0021 in CD19^+^ B cells). Figure 3 illustrates the methylation patterns of a highly monocyte-specific probe versus a probe showing a similar trend across cell types. Violin plots of uncorrected beta values for the remaining significant probes are shown in Supplementary Fig. 4. We noted an unusual distribution of methylation levels for two of the probes, cg18523915 and cg14704780, showing particularly high variability in healthy controls. This might indicate that some unknown external factor is having a major impact on methylation at these CpGs, potentially confounding our association results. Cg18523915 has been reported to be significantly associated with outcome of COVID-19 in two recent EWAS studies of whole blood^35^ and peripheral blood mononuclear cells (PBMCs)^36^, yet blood sampling in our study was performed prior to the COVID-19 pandemic.

We compared our results to the two largest whole-blood EWAS published in refs. ^30^ and Henderson-Smith et al. (Supplementary Table 2). The two significant probes reported by Vallerga et al. showed no evidence of association in any cell type in our data. Conversely, we looked up the seven significant probes from the present study in whole-blood summary statistics and found that three are present in the Vallerga et al. data, yet none showed evidence of association with PD in whole-blood data. Out of the seven probes reported as significantly associated with PD in whole blood by Henderson-Smith et al., the two top probes showed associations at *P* < 0.05 with one cell type each (cg06889422 in CD4^+^ T cells, *P* = 0.042, and cg16133681 in CD14^+^ monocytes, *P* value = 0.042), both with direction of effect consistent with the whole-blood study (Supplementary Table 2). None of the previously reported probes showed association in any of the four immune cell types when adjusting for 36 independent lookups.

We further compared the genomic location of the seven significant probes to the top signals reported in the largest PD GWAS to date^9^. One CpG probe, cg14704780 annotated to *TSBP1*, is located less than 300 kb from the top GWAS SNP of the HLA locus, rs112485576. No other CpG probe fell within a 1-Mb window of any GWAS top SNP. We assessed methylation quantitative trait loci (mQTL) associated with cg14704780, cg20636526 or rs112485576 in whole blood using the ARIES mQTL database^37^, but found no data to support a significant association between the GWAS SNP and differential methylation at these particular CpGs.

### Differentially methylated regions and gene ontology enrichment in CD14^+^ monocytes

*P* value histograms and individual CpG association results clearly suggested that our experiment had the best potential to detect true signals in CD14^+^ monocytes, and we prioritized this cell type for analysis of differentially methylated regions. The DMRcate package estimates a smoothed test statistic across probes within a given window and compares this to the expected statistic, using the Benjamini–Hochberg false discovery rate method for calling individually associated probes. A more liberal significance threshold for this initial step than the Bonferroni correction used in the individual CpG association analysis has been recommended^38^, as the method requires multiple associated probes in the same region, which is also more likely to be biologically relevant than single associated probes. The method identified four differentially methylated regions at *P* < 0.05 based on Fisher’s multiple comparison statistic, all in CD14^+^ monocytes (Table 3). Among the significant individual CpGs in monocytes were three hypomethylated probes located near *LINC03062* on chromosome 9 and 1 hypermethylated probe annotated to *TSBP1*. These were also represented in the two most significant regions (chr9: 92,276,541–92,277,266 and chr6: 32,305,106–32,305,145) identified by DMRcate. The next region at chr6: 146,863,357–146,863,680 is located upstream of *RAB32* and includes three hypomethylated probes that did not reach individual significance at the Bonferroni-corrected threshold. Gene ontology pathway analysis using *gometh* (see “Methods”) did not identify any significantly enriched GO or KEGG pathways, which was not unexpected given the limited number of significant probes identified for each cell type.

**Table 3:** Differentially methylated regions in CD14^+^ monocytes.

Region (b37)	N CpGs	*P* value (Fisher)	Closest gene
chr9: 92,276,541–92,277,266	4	2.14e-10	*LINC03062*
chr6: 32,305,106–32,305,145	2	1.94e-5	*TSBP1*
chr6: 146,863,357–146,863,680	3	0.015	*RAB32*
chr10: 105,428,385–105,428,818	5	0.032	*SH3PXD2A*

Differentially methylated regions were analyzed using the DMRcate R package with default parameters. *P* values correspond to Fisher’s multiple comparison statistic, as recommended in the DMRcate documentation.

### Comparison with monocyte gene expression and qPCR analysis of *RAB32*

A predominance of significant findings in monocytes is in line with previous research implicating the myeloid lineage in PD. A recent study investigated monocyte gene expression in 135 PD patients and 95 controls and identified 300 differentially expressed genes^15^. Comparison with our results is not straightforward, as differential methylation analysis identifies regulatory genomic loci rather than genes. Mindful of this caveat, we performed a lookup of the closest gene to each of the differentially methylated probes and regions in monocytes (ten different genes in total) in the summary statistics from differential gene expression provided by Navarro et al. Applying Bonferroni correction for ten independent tests, *RAB32* was the only differentially expressed gene (*P* value = 0.0012), upregulated in PD. We also compared the genes proximal to differentially methylated loci in monocytes to 29 genes previously highlighted in a transcriptome-wide association study (TWAS) using gene expression models from peripheral monocytes but found no overlap with our ten monocyte signals^39^.

**Fig. 4:**
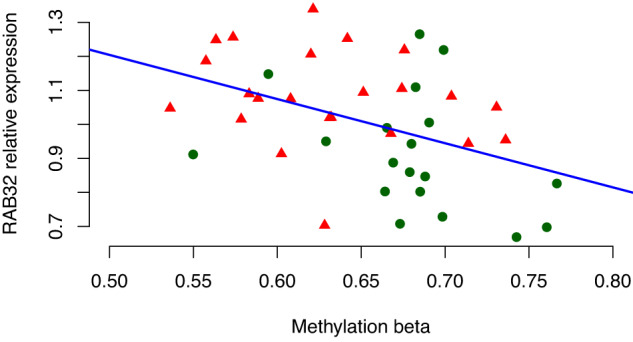
Scatterplot of cg05420134 and *RAB32* expression. The plot illustrates the correlation between cg05420134 methylation beta values and *RAB32* mRNA expression assessed by quantitative PCR. PD patient data points are shown as triangles (red) and control data points as circles (green). Expression values are calculated as relative to the mean. The regression line indicates the coefficient (SD) = − 1.30 (0.43).

To further assess whether differential methylation near *RAB32* was also reflected on the mRNA level in our monocyte samples, we performed qPCR (see “Methods”). In line with the results from Navarro et al., we found that *RAB32* was upregulated in PD compared to controls (*P* value = 0.0020, effect size of disease status on expression Z score (SD) = 0.93 (0.28)). We further observed a strong negative correlation between methylation and *RAB32* expression (Pearson’s *r*^2^ = −0.43, *P* value = 0.0045) (Fig. 4) Taken together, these results indicate that PD patients show hypomethylation upstream of *RAB32* combined with increased *RAB32* expression in monocytes.

### Exploring the association between differentially methylated probes and dopaminergic treatment

DNA methylation may be altered by medical treatment, and previous work has indicated that this may be relevant in PD^40^. Aiming to explore the likelihood that observed differences in methylation may be driven by treatment effects, we assessed the association between all differentially methylated loci and dopaminergic medication as a binary (treatment-naïve versus on PD medication) variable. No association passed adjustment for multiple testing, but we observed *P* value < 0.05 for the probe in the *TSBP1* locus of hypomethylation with dopaminergic treatment (coefficient = −0.60 (0.20), *P* value = 0.0062 for association between cg14704780 and treatment). Interestingly, this indicates that methylation levels differed more from controls in treatment-naïve patients than in patients receiving medication, a pattern also reported previously for *SNCA* promoter methylation in whole blood^40^. We note however, that the level of motor symptoms as assessed by the Unified Parkinson’s Disease Rating Scale (UPDRS) III was significantly higher in the patients who had not yet started PD treatment (Wilcoxon rank sum test *P* value = 0.02). The association between 14704780 and dopaminergic treatment was weakened by including UPDRS III in the model, but not eliminated (*P* value = 0.014).

## Discussion

There is an urgent need to elucidate the immune mechanisms that contribute to PD pathogenesis. We performed a hypothesis-free methylome-wide association study of purified immune cell populations in early PD, identifying differential methylation across ten different genomic loci in CD14^+^ monocytes, and one in CD8^+^ T cells. Our findings indicate dysregulation of immune cells in PD, showing cell-type-specific changes that would not be readily detectable by whole-blood methylation analyses. We included PD patients with less than five years disease duration from diagnosis, 1.2 years on average, indicating that these methylation changes are present early in the disease course. Of the four different immune cell types studied, CD14^+^ monocytes showed by far the strongest evidence of differential methylation, both as individually significant probes and differentially methylated regions identified by DMRcate. An overrepresentation of low *P* values at the tail of an otherwise uniform distribution was seen only in CD14^+^ monocytes, which might potentially reflect more true disease-associated methylation changes in this cell type. These observations are in line with an increasing body of evidence implicating the myeloid lineage in neurodegeneration generally^10,11^, as well as in PD specifically^13,15,39^.

A differentially methylated region with three individually significant probes was identified in monocyte data near the long noncoding RNA *LINC03062*, alternative name *UNQ6494*, on chromosome 9. This transcript has been linked to immune infiltration in endometrial carcinoma^41^ and survival in lung adenocarcinoma^42^, but potential roles in neuroinflammation are unknown. The region is located ~50 kb downstream of its closest coding gene, *GADD45G*, which has functions related to DNA damage response and cellular stress signaling^43^.

We also identified a hypomethylated region in monocytes upstream of *RAB32* and found by qPCR that *RAB32* monocyte expression both correlated negatively with methylation in this region and was increased in PD patients relative to controls. Independent evidence supporting a role for this gene was found in summary statistics from a recently published transcriptomic study, also indicating increased expression in PD monocytes^15^. Interestingly, *RAB32* encodes a small Rab GTPase that has been demonstrated to directly interact with and regulate leucine-rich repeat kinase 2, *LRRK2*^44–46^. Coding mutations in *LRRK2* cause autosomal dominant PD^47,48^ and noncoding common variants in the same locus are associated with sporadic PD in GWAS^9^. Several lines of evidence link the pathogenic mechanisms of *LRRK2* to cells of the myeloid lineage, potentially involving both microglia^49,50^ and monocytes^15,51^. Our findings thus provide further support for *RAB32* as implicated in PD pathogenesis through interaction with *LRRK2* in myeloid cells.

Comparing the methylation patterns of significant probes across all cell types revealed that the differential methylation was largely cell-type-specific. Furthermore, we observed very limited overlap between our own findings and results from recent PD EWAS of whole blood^28,30^. It is well established that PD patients have increased levels of granulocytes and decreased levels of lymphocytes compared to healthy controls^52,53^, and although algorithms for cell-type deconvolution are used, there is always the risk that cell composition may be affecting the results. Even if the adjustment for cell composition is accurate, the methylation measured in whole blood will be a mixture reflecting all cell types, quantitatively dominated by granulocytes. Our findings indicate that isolation of homogenous cell populations is essential to identify disease-relevant methylation changes in less abundant cell types. Fluorescence-activated cell sorting (FACS) is an alternative to isolation with magnetic beads used here, and we acknowledge that processing a subset of samples with both methods would have allowed for further methodological validation of this step. Of note, RNA sequencing is increasingly being applied at single-cell resolution, yet this methodology is generally not yet available for DNA methylation analysis.

Our study has several limitations. Most importantly, there is a lack of independent replication of the results. The findings must therefore be interpreted with caution until they can be corroborated by similar methylation analyses from other cohorts. In our attempt to find independent support for 11 genes nominated in monocytes by comparison with summary statistics from a monocyte differential expression analysis reported in a recent PD study^15^, *RAB32* was the only gene passing an adjusted significance threshold. This strengthens our report of hypomethylation upstream of *RAB32* yet limits the interpretability of other signals. It is worth noting however, that simple one-to-one relationships between methylation and gene expression are not the rule in analyses incorporating both types of data^54^.

DNA methylation studies cannot differentiate between disease causes and downstream effects, the latter being less relevant for translational research aiming toward novel disease-modifying therapies. We included patients in an early phase of the disease, including nearly one-third de novo patients who had not started medication for PD. Nevertheless, we cannot exclude that treatment effects or other disease-related effects without causal relevance may have contributed to the observed methylation changes. We observed a trend towards association with medication for probes annotated to *TSBP1*, which were also located close to the established PD GWAS signal in the HLA region, but where treatment seemed to associate with a normalization of methylation values.

Importantly, a wide range of lifestyle and environmental factors may differ across groups of PD patients and healthy controls, potentially influencing DNA methylation. Such exposure-driven methylation differences may be mediators of PD risk, but could also be by-products of risk-associated exposures with no causal role or downstream effects of living with PD. Our study did not include the rich exposure data that would be required to dissect these complex relationships. This limitation is a major general caveat for most current complex disease EWAS, however. As highlighted in a recent review, improved integration of exposure variables is highly warranted in future epigenetic studies of PD^55^.

The included number of participants (25 patients and 25 controls) was modest, although we analyzed a total of 186 methylomes across four cell types. Power analysis indicated that our design was underpowered for smaller effect sizes, which we recognize as a major limitation of our study. *P* value histograms suggested that the data would have been better modeled in sex-stratified analysis for CD8^+^ T cells and CD19^+^ B cells, yet our study was too small to identify significant signals using this design. Power was also limited for pathway analyses, and larger studies are needed to further characterize methylation changes across the studied cell types. Furthermore, this study was performed in an ethnically homogeneous European population. This is a strength with respect to the marginal statistical power, yet do not know to what degree disease-associated methylation changes tend to be population-specific. As with genetic association studies, we acknowledge that more research in underrepresented populations is urgently needed.

In conclusion, we performed a methylome-wide association study of purified CD14^+^ monocyte, CD19^+^ B cell, CD4^+^ T-cell and CD8^+^ T-cell populations in PD, identifying differential methylation predominantly in CD14^+^ monocytes, highlighting the *RAB32* locus in particular. Our findings shed further light on immune mechanisms in PD and warrant further studies of these cell types in order to clarify their role in pathogenesis and identify potential targets of immune-modifying therapy.

## Methods

### Subjects

The project was approved by the Regional Committee for Medical and Health Research Ethics, Norway. Participants were recruited at the Department of Neurology, Oslo University Hospital and gave written informed consent. A diagnosis of PD was made based on the Movement Disorder Society criteria^31^, yet levodopa response was not required for inclusion of treatment-naïve patients. Subjects with immunological disorders were excluded, and a clinical blood screen was performed to confirm no gross abnormalities in leukocyte counts at the time of blood draw.

### Isolation of immune cell types

Peripheral blood mononuclear cells (PBMC) were isolated from whole blood immediately after venipuncture by Lymphoprep (Stemcell Technologies, Vancouver, Canada) and density gradient centrifugation. Next, CD8^+^, CD14^+^ and CD19^+^ cells were isolated by positive selection with EasySep^TM^ Human CD8/CD14/CD19 Positive Selection Kit II (Stemcell Technologies, Vancouver, Canada). CD4^+^ cells were isolated by negative selection with EasySep^TM^ Human CD4 Isolation Kit (Stemcell Technologies, Vancouver, Canada). All procedures followed the standard protocol from the kit manufacturer. Flow cytometry was performed as a quality control the next day to ensure a purity of >90% for each isolated cell population.

### Power analysis

We estimated power using the online tool pwrEWAS^32^. The number of CpGs tested was set to 500,000, target CpGs to 50 and false discovery rate to 0.05. Based on these parameters, a study of 25 patients and 25 controls was estimated to have 80% power to detect a differential methylation corresponding to 0.1 SD. Assuming a replication scenario where 10 candidate CpGs are tested, out of which 5 are truly associated, the required effect size for 80% power was 0.028 SD.

### DNA methylation analyses, quality control, and data normalization

DNA from the four cell types was isolated with QiAamp DNA Mini Kit (Qiagen, Hilden, Germany) using the standard protocol from the manufacturer. DNA isolation yield varied across samples. From a total of 50 participants, a sufficient amount of DNA was available for 49 CD14^+^ samples, 50 CD19^+^ samples, 49 CD8^+^ samples, and 38 CD4^+^ samples. Insufficient DNA yield occurred primarily for CD4^+^ T cells, at a similar rate in patients (5/25) and controls (7/25). 500ng DNA from each sample was bisulfite treated and assessed using the Illumina Infinium MethylationEPIC BeadChip (Illumina, San Diego, CA). Samples of different disease status and cell type were deliberately intermixed on plates and arrays in a balanced design. From four of the samples, we included an identical technical DNA replicate in the experiment for quality check purposes. The MethylationEPIC array targets ~850,000 CpGs across the regulatory human genome, including both promoter regions and distal enhancers.

Data processing was performed using R 4.0.3. Raw signal intensity data were imported into R before applying a series of quality checks and filtering steps implemented in the minfi^56^ and wateRmelon^57^ R packages. Quality control, normalization and evaluation of potential batch effects were performed jointly on the full experiment dataset including all cell types. One sample where >1% of sites had a detection *P* value greater than 0.05 was filtered out using the *pfilter* function in the wateRmelon package. CpG sites with a beadcount <3 in 5% of samples or detection *P* value > 0.05 in 1% of samples were also filtered out. Sex chromosome CpGs were used to estimate sample sex and one sample failing sex check was removed. All remaining data were of high quality as evaluated by inspection of bisulfite conversion and other control probe metrics, outlier detection by the wateRmelon *outlyx* function and assessment of median signal intensities as evaluated by the minfi *getQC* function. From a broad range of available normalization methods, we chose functional normalization (*funnorm*) implemented in the minfi package, as this method is recommended when large differences in methylation patterns are expected, including data from different cell types^58^. We note that although assessment of differential methylation within each cell type was the main aim of the study, appropriate evaluation of potential batch effects required reliable comparison also across the different cell types.

The normalized *MethylSet* data object was mapped to the genome and probes on sex-chromosomes, probe sequences overlapping with known SNPs in the MethylationEPIC annotation and previously reported cross-reactive probes^59^ were filtered out. Taking advantage of the technical replicates we used the CpGFilter package to compute the intra-class correlation coefficient (ICC), which characterizes the relative contribution of the biological variability to the total variability for each probe^60^. Probes with low ICC have large measurement errors, making them unsuitable for statistical association testing in complex disorders, and a considerable proportion of probes may thus be filtered out, reducing the multiple testing burden. We filtered out probes with ICC < 0.2. Furthermore, we filtered out constitutively methylated or unmethylated probes with mean beta values < 0.025 or >0.975. The wateRmelon *pwod* function was used separately on data from each cell type to filter out outlier values lying more than four times the interquartile range from the mean, assumed to result from rare SNP artifacts.

### RAB32 expression analysis with quantitative PCR

RNA was extracted from CD14^+^ monocytes using the RNeasy Mini Kit (Qiagen, Hilden, Germany), with adequate yield for 23 PD and 20 control samples, followed by cDNA synthesis using SuperScript IV VILO Master Mix with ezDNase (Invitrogen, Waltham, MA). Quantitative PCR (qPCR) was performed using TaqMan gene expression assays on a ViiA7 instrument (Applied Biosystems, Waltham, MA) with standard settings as recommended by the manufacturer. Based on previous literature^61,62^, we evaluated *ACTB*, *RPL37A* and *B2M* as reference genes and selected *RPL37A* and *B2M* as the most suitable based on a strong pairwise correlation (Pearson’s *R*^2^ = 0.77, *P* value = 1.06e-9) compatible with stable expression. The geometric mean of the cycle threshold (CT) value for these genes was used as normalization factor to estimate the relative expression of *RAB32* using the comparative CT method^63^.

### Statistical analyses

Methylation array experiments are prone to technical batch effects. We evaluated experiment plate and chip position as potential covariates but found that these showed no association with top five principal components. To adjust for potentially unknown batch effects, we estimated surrogate variables (SVs) using the sva package^64^. We applied the “Leek” method to determine the number of SVs to include in the model^65^, which identified 1 SV as appropriate. Methylation beta values were logit transformed into *M* values. To identify differentially methylated sites (CpGs), linear regression was performed using the limma package^66^, using the *makeContrasts* function to define the contrast of interest between PD and control samples of each cell type. We included sex, age, and the first SV as covariates in the model: M ~ disease status + age + sex + SV1. We used the Bonferroni-corrected significance threshold recommended by Mansell et al.^67^ (*P* < 9 × 10^–8^) divided by four cell types (*P* < 2.25 × 10^–8^) to adjust for multiple testing. To assess differentially methylated regions, we used the contrast matrix generated for linear regression as input for the R package DMRcate with default parameters^68^. Finally, we assessed gene ontology enrichment of significant CpGs using the *gometh* function implemented in the missMethyl R package^69^. In CD14^+^ monocytes we tested both Gene Ontology (GO) and Kyoto Encyclopaedia of Genes and Genomes (KEGG) pathways for CpGs passing significance thresholds of *P* value < 10^–7^, as well as a more liberal threshold of *P* value < 10^–4^. We explored the association between significant probes and dopaminergic treatment using linear regression, comparing treated (*N* = 17) to untreated (*N* = 8) patients with sex, age and one the first surrogate variable as covariates.

The correlation between *RAB32* expression and adjacent CpGs was investigated using Pearson’s product-moment correlation. Linear regression with sex and age as covariates was used to assess the association between *RAB32* expression and disease status. After the identification of differentially methylated CpGs and regions, we assessed the potential association of these probes with PD medication and clinical outcomes using linear or logistic regression models as appropriate, with sex and age as covariates.

### Reporting summary

Further information on research design is available in the Nature Research Reporting Summary linked to this article.

### Supplementary information

Suppmental material

Reporting Summary

## Data Availability

The individual-level raw data generated in the current study are not publicly available as open sharing was not included in the informed consent signed by the participants. Data will be provided from the corresponding author for noncommercial PD research use provided a collaboration and data transfer agreement has been signed and approved by the institutional Data Protection Office. Full summary statistics will be made publicly available at https://github.com/lpihlstrom.

## Code availability

The underlying software code for this study will be made fully available on GitHub at https://github.com/lpihlstrom.
