# Supplementary material for: Epigenome-wide association study of peripheral immune cell populations in Parkinson’s disease
Source: NPJ Parkinsons Dis. 2023 Oct 31;9:149. doi: 10.1038/s41531-023-00594-x (PMC10616224; doi:10.1038/s41531-023-00594-x)
Supplement: Supplementary file 1 — Suppmental material [file 41531_2023_594_MOESM1_ESM.pdf]

Supplement to:

Epigenome-wide association study of peripheral immune cell populations in Parkinson's disease

Maren Stolp Andersen, Ingvild Sørum Leikfoss, Ina Skaara Brorson, Chiara Cappelletti, Conceicao Bettencourt, Mathias Toft and Lasse Pihlstrøm*

* Corresponding author at: lasse.pihlstrom@medisin.uio.no

Supplementary figure 1. Data processing pipeline

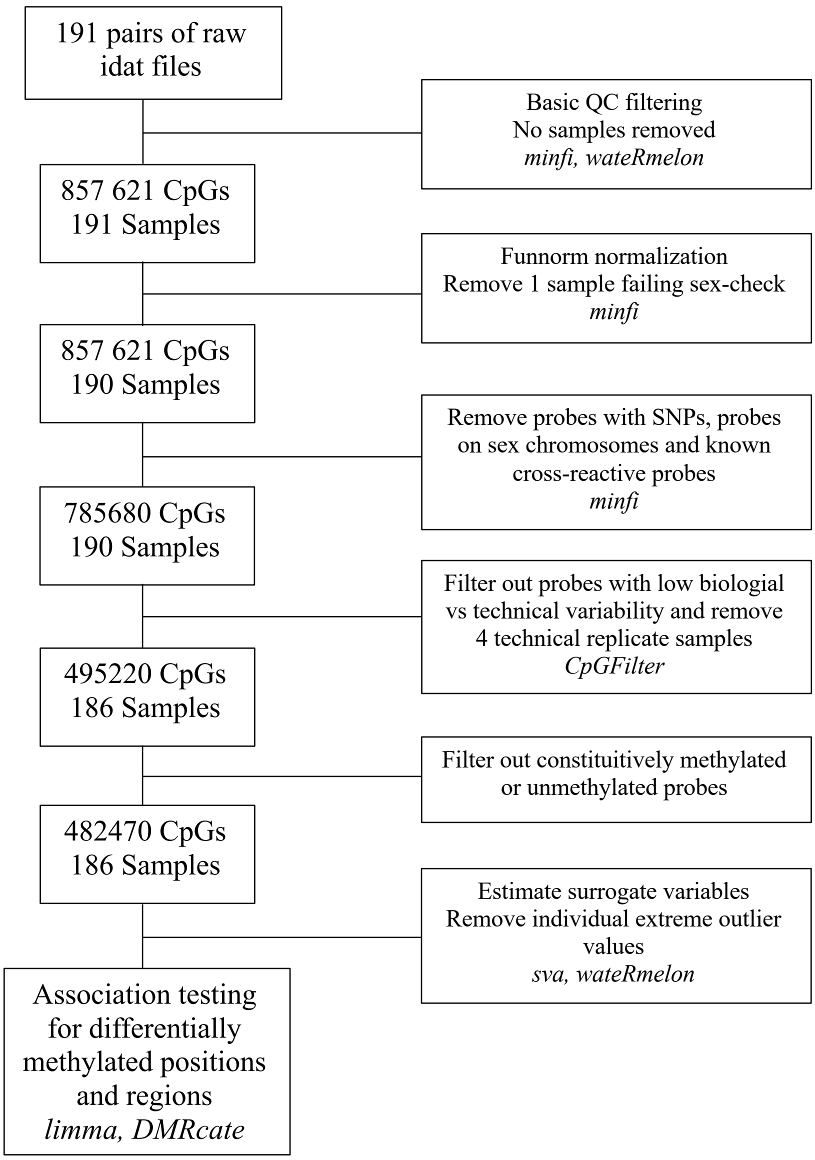

Outline of quality control and filtering pipeline from raw data to differential methylation analysis. R packages used at each step are indicated in *italics*.

Supplementary figure 2. Quantile-quantile plots

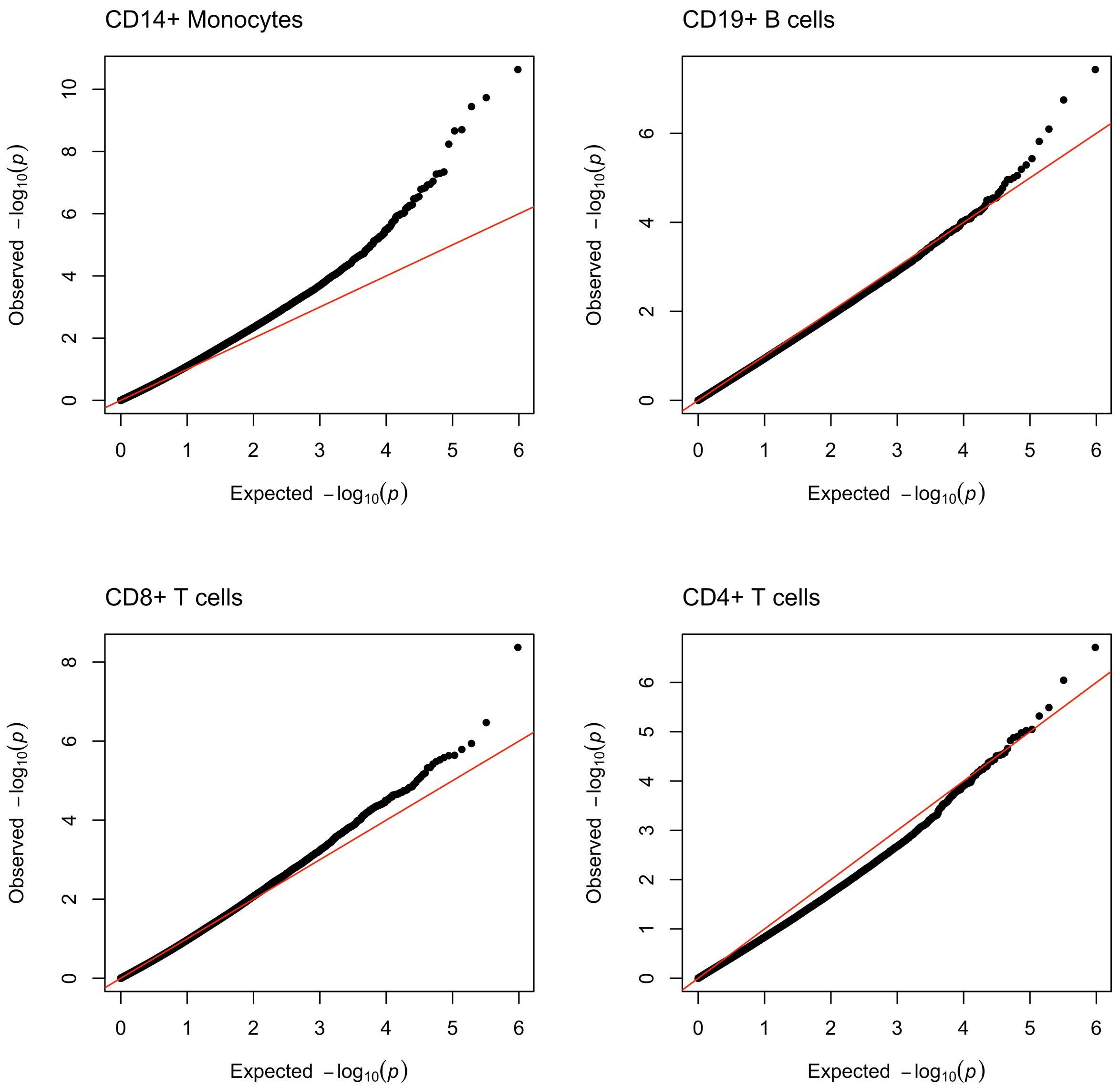

Quantile-quantile plots from linear regression analyses of differentially methylated positions, including sex, age and the first surrogate variables.

Supplementary Figure 3. P-value histograms

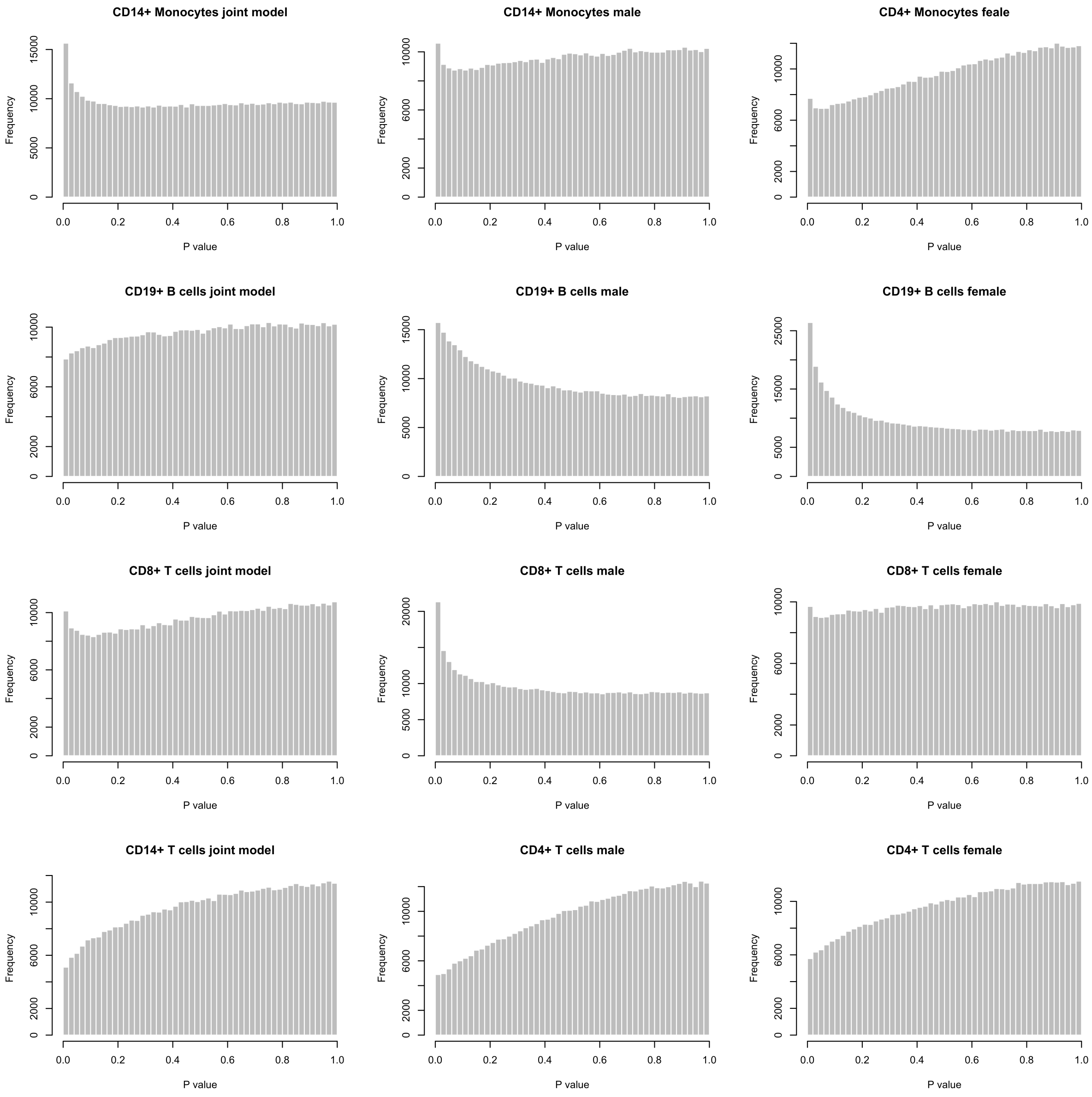

Histograms showing the distribution of p-values for linear regression in each cell type, comparing the main model using sex as a covariate (left) to sex-stratified analyses for males (middle) and females (right) separately. Note the improved p-value distribution in sex-stratified analysis for CD19+ B cells and CD8+ T cells for males.

Supplementary Figure 4. Violin plots

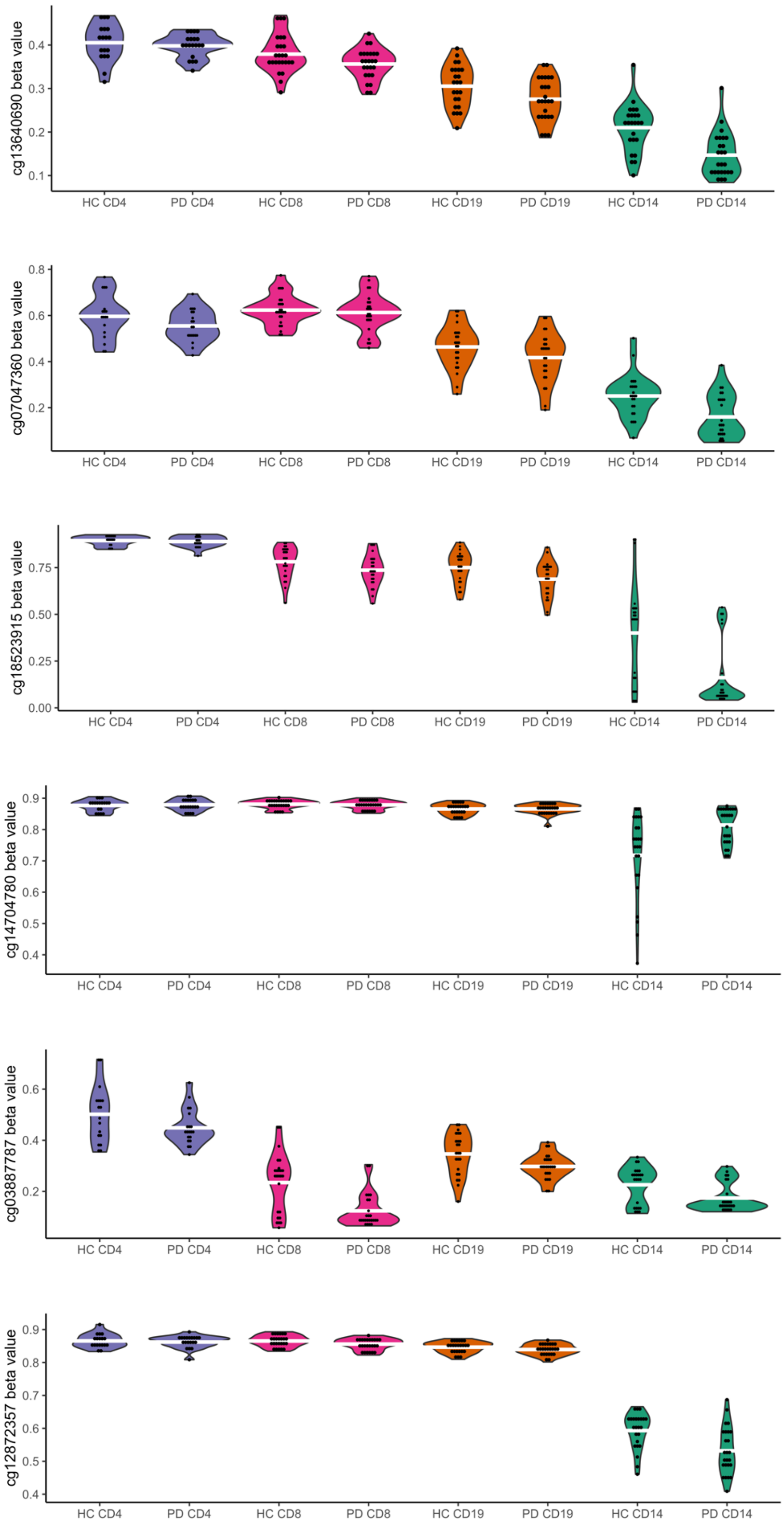

Violin plots showing uncorrected beta values for significant CpGs, and cg12872357 from the differentially methylated region near *RAB32*.

Supplementary Table 1. Association statistics for significant probes across all cell types

Probe	CD14 coeff	CD14 SE	CD14 P	CD19 coeff	CD19 SE	CD19 P	CD4 coeff	CD4 SE	CD4 P	CD8 coeff	CD8 SE	CD8 P
cg13640690	-0.68	0.10	2.32E-11	-0.25	0.10	0.010	-0.12	0.11	0.258	-0.19	0.10	0.048
cg12134806	-0.68	0.10	1.85E-10	-0.17	0.10	0.094	-0.13	0.12	0.270	-0.12	0.10	0.224
cg11473614	0.44	0.07	3.60E-10	0.21	0.07	0.002	0.15	0.08	0.053	0.16	0.07	0.020
cg07047360	-1.03	0.16	1.99E-09	-0.37	0.16	0.025	-0.42	0.19	0.027	-0.18	0.16	0.278
cg18523915	-1.98	0.32	2.18E-09	-0.40	0.32	0.201	-0.01	0.36	0.982	-0.28	0.31	0.366
cg14704780	0.72	0.12	5.81E-09	0.00	0.12	0.976	-0.02	0.14	0.911	-0.04	0.12	0.723
cg03887787	-0.45	0.17	0.008	-0.29	0.17	0.087	-0.40	0.19	0.042	-1.03	0.17	4.27E-09

The table shows association statistics from linear regression across the 4 investigated cell types for each of the probes that passed a Bonferroni-corrected significance threshold in one cell type.

Supplementary Table 2 Summary statistics on probes reported in whole blood PD EWAS

Probe	Reported in whole blood by	Direction WB	CD14 coeff	CD14 SE	CD14 P	CD19 coeff	CD19 SE	CD19 P	CD4 coeff	CD4 SE	CD4 P	CD8 coeff	CD8 SE	CD8 P
cg06690548	Vallerga et al.	-	0.07	0.10	0.49	0.11	0.11	0.30	0.06	0.12	0.60	0.10	0.10	0.31
cg12724357	Vallerga et al.	-	-0.20	0.15	0.19	-0.29	0.15	0.06	0.02	0.18	0.93	-0.11	0.15	0.48
cg06889422	Henderson-Smith et al	-	-0.13	0.10	0.20	-0.02	0.10	0.81	-0.23	0.11	0.04	-0.11	0.10	0.26
cg16133681	Henderson-Smith et al	-	-0.37	0.18	0.04	-0.09	0.18	0.63	-0.27	0.21	0.19	-0.04	0.18	0.83
cg26524067	Henderson-Smith et al	-	-0.06	0.21	0.77	-0.24	0.21	0.26	-0.11	0.24	0.65	-0.06	0.21	0.77
cg09994891	Henderson-Smith et al	+	0.08	0.13	0.54	0.06	0.13	0.65	-0.11	0.15	0.48	-0.09	0.13	0.52
cg11408952	Henderson-Smith et al	+	0.19	0.24	0.43	0.42	0.24	0.08	-0.01	0.29	0.96	0.08	0.24	0.73
cg26033520	Henderson-Smith et al	+	-0.06	0.19	0.74	0.11	0.19	0.55	0.01	0.22	0.96	0.22	0.19	0.25
cg08098382	Henderson-Smith et al	+	0.09	0.12	0.48	0.06	0.12	0.61	0.02	0.14	0.88	0.15	0.12	0.23

The table shows association statistics from linear regression across all 4 investigated cell types for 2 probes reported by Vallerga et al. and 7 probes reported by Henderson-Smith et al. in whole blood EWAS of PD.
